# A Preliminary Report of A Low-Dose Step-Up Regimen of Recombinant Human FSH for Young Women Undergoing Ovulation Induction with IUI

**DOI:** 10.22074/ijfs.2015.4600

**Published:** 2015-12-23

**Authors:** Hsin-Fen Lu, Fu-Shiang Peng, Shee-Uan Chen, Bao-Chu Chiu, Szu-Hsing Yeh, Sheng-Mou Hsiao

**Affiliations:** 1 Department of Obstetrics and Gynecology, Far Eastern Memorial Hospital, New Taipei, Taiwan; 2 Department of Obstetrics and Gynecology, National Taiwan University Hospital, Taipei, Taiwan

**Keywords:** Infertility, Ovarian Hyperstimulation Syndrome, Ovulation Induction

## Abstract

**Background:**

The aim of this study was to evaluate the efficacy and safety of a recombinant human follicle stimulating hormone (r-FSH) low-dose step-up regimen for controlled ovarian hyperstimulation in patients undergoing ovulation induction (OI) with intrauterine insemination (IUI).

**Materials and Methods:**

The study was conducted in the Department of Obstetrics and Gynecology, Far Eastern Memorial Hospital, New Taipei, Taiwan. In this prospective, observational study, consecutive infertile women (20-35 years) with regular menstrual cycles and a normal baseline FSH level were prospectively enrolled between January 2010 and September 2010. A starting dose of 112.5 IU/day r-FSH was administered on day 3 and increased by 37.5 IU/day every 2 days until a follicle ≥11 mm in diameter was present. Recombinant human chorionic gonadotropin (r-hCG) was administered when a follicle ≥18 mm was noted. Monifollicular development was defined as only one follicle with a diameter ≥16 mm. Clinical pregnancy was defined as a pregnancy diagnosed by ultrasonographic visualization of one or more gestational sacs.

**Results:**

A total of 29 women and 30 cycles were included. The mean daily dose of r-FSH to achieve a follicle of ≥11 mm in diameter was 131.3 ± 23.6 IU and the mean total dose was 1030.0 ± 383.2 IU. Approximately 41% of the cycles were monofollicular. Clinical pregnancy was observed in 9 (30.0%) cycles, and a fetal heart beat was observed in 7 (23.3%). There were no multiple pregnancies. Mild ovarian hyperstimulation syndrome, which was resolved with conservative management, was observed in 3 (10.0%) cycles.

**Conclusion:**

This r-FSH low-dose step-up regimen seems to be a feasible and practical method for OI in younger infertile women undergoing IUI.

## Introduction

Controlled ovarian hyperstimulation improves the cycle fecundity rate in part by increasing the number of follicles available for fertilization and correcting subtle, unpredictable ovulatory dysfunction. Intrauterine insemination (IUI) is an established treatment for infertility due to cervical factor, male factor, or with an unexplained etiology. Combined with IUI, ovulation induction (OI) is recommended for many causes of infertility in patients with patent fallopian tubes (1). The overall success rate of IUI is approximately 10-15% (2,3). Women with minor endometriosis or infertility due to unknown reasons may elect to undergo OI/ IUI to increase the pregnancy rate, but the risk of multiple gestations is also increased (3). To date, no ideal stimulation protocol that provides a high pregnancy rate and low rate of complications such as ovarian hyperstimulation syndrome (OHSS) and multiple pregnancies has been identified (3,4). 

OI aims at the selection of a single follicle that will be able to reach the pre-ovulatory size and rupture. A recent retrospective cohort study indicated that induction of more than one follicle did not improve the ongoing pregnancy rate, but increased the risk of multiple pregnancies (3). Thus, it was suggested that in all IUI cycles for unexplained non-conception monofollicular growth should be sought to reduce the number of multiple pregnancies. Controlled OI with recombinant human follicle stimulating hormone (rFSH) has been shown to be predictive of ongoing pregnancy rate, and studies have attempted to determine the r-FSH threshold (i.e. r-FSH dose on the day when a follicle is <10 mm in diameter) on the basis of pre-treatment and screening characteristics (4,6). The Gonal-f ^®^New Generation Pre-Filled Pen (Merck Serono, Germany) is a disposable, pre-filled drug delivery system intended for the subcutaneous injection of multiple and variable doses of a liquid formulation of r-FSH. It is indicated to induce the development of multiple follicles in patients participating in an assisted reproductive technology program (7,10). Though limited data is available, studies have also shown that r-FSH seems to be at least as effective as urinary FSH preparations (11,13) and exhibits a similar safety profile (11). 

Because the induction of more than one follicle does not increase the pregnancy rate, but does increase the risk of multiple gestations, the r-FSH threshold that can result in monofollicular development should be identified. The purpose of this study was to evaluate the safety and efficacy of an r-FSH low-dose step-up regimen for OI/IUI. 

## Materials and Methods

This was a single center, prospective, observational study on the use of r-FSH ( Gonal-f ^®^ in subjects undergoing OI/IUI. All patients provided written informed consent for participation in the study, and the study was approved by the Research Ethics Committee of Far Eastern Memorial Hospital, New Taipei, Taiwan. 

The inclusion criteria were women between 20 and 35 years of age, regular menstrual cycles of 25-35 days, the presence of both ovaries, normal uterine cavity and patent fallopian tubes as investigated by either ultrasound scan, hysteroscopy, or hysterosalpingography, normal baseline serum FSH level (<10 μg/dL), and male partner semen analysis considered adequate for IUI in accordance to the center’s standard practice (i.e. <1×10 ^7^ sperm/mL after sperm washing). The exclusion criteria included extrauterine pregnancy or abortion in the past 3 months, abnormal gynecologic bleeding of undetermined origin, history of OHSS, and known hypersensitivity to human rFSH preparations. 

The objective of r-FSH therapy is to develop a single mature Graafian follicle from which the ovum will be liberated after the administration of hCG. A starting dose of r-FSH 112.5 IU/day was begun on the 3^rd^ day following an induced or spontaneous menses. Transvaginal ultrasound (SSD-1700, Aloka Co., Japan) monitoring was performed every 2 days beginning on the 7^th^ day. If on the 7^th^ day, a follicle had not reached 11 mm in diameter, the dose was increased to 150 IU/day (+37.5). If ultrasound on the 9th day did not show a follicle had reached 11 mm, the dose was again increased by 37.5 IU (187.5 IU/day). The same increase was made if on the 11th day, a follicle had not reached 11 mm. The maximum dose administered was 225 IU/day.

When an optimal response was obtained (dominant follicle ≥18 mm), a single subcutaneous injection of recombinant-human chorionic gonadotropin (r-hCG, 6500 IU, Ovidrel®, Merck Serono, Germany) was administered 24 hours after the last r-FSH injection. Serum estradiol (E_2_) was measured on the day of rhCG. Intrauterine insemination was then performed 24 hours later. If an excessive ovarian response (i.e. E_2_>3500 μg/dL) occurred, treatment was stopped and r-hCG withheld. A new cycle was then initiated at a lower r-FSH dosage than that of the prior cycle, and the cycle with the hyper-response was excluded from the analysis. A maximum of three cycles (excluding those in which a hyper-response occurred) were allowed in an individual patient.

Monifollicular development was defined as only one follicle with a diameter ≥16 mm. Clinical pregnancy was defined as a pregnancy diagnosed by ultrasonographic visualization of one or more gestational sacs based on the definition proposed by the International Committee for Monitoring Assisted Reproductive Technology (ICMART) (14). 

### Statistical analysis

The primary endpoint was the clinical pregnancy rate. The secondary endpoints were multiple pregnancy rate and occurrence of OHSS. The Shapiro-Wilk test was implemented to test whether the distributions of continuous variables met the assumption of a bell shape. Normally distributed continuous data were presented as mean ± standard deviation (SD), while categorical data were presented as number (n) and percentage (%). Nonnormally distributed data were presented as median (range). Descriptive statistics were performed using Statistical Package for the Social Sciences (SPSS) version 15.0 (SPSS Inc., USA). 

## Results

Between January 2010 and September 2010, 30 consecutive women were enrolled in the study. The study was conducted in the Department of Obstetrics and Gynecology, Far Eastern Memorial Hospital, New Taipei, Taiwan. One patient with an abnormal baseline serum FSH level was excluded in the follow- up visit. Twenty-eight women underwent only one OI/IUI cycle. One woman failed to get pregnant at her first OI/IUI cycle, and then received her 2nd OI/ IUI cycle. Thus, a total of 30 OI/IUI cycles were performed in this study and analyzed.

Baseline data are shown in table 1. On the day of r-hCG, all 30 cycles had follicles at least 16 mm in diameter, the median E_2_level was 898.0 pg/mL, and the mean endometrial thickness was 12.0 mm. The average total r-FSH dose was 1030.0 IU, and the average daily r-FSH dose was 122.5 IU. The average r-FSH dose when the follicular diameter was <10 mm in diameter (i.e. threshold of r-FSH) was 131.3 IU. The average length of time from the first injection of r-FSH until the day of r-hCG injection was 8.0 days. Twelve cycles met the defined criteria for monofollicular development on the day of r-hCG administration (Table 2). 

Clinical pregnancy was observed in nine ( 30.0%, 95% confidence interval (CI)=12.6 to 47.4%) cycles, and a total of nine gestational sacs were found at follow-up. However, lack of fetal heart activity was found in two gestational sacs, thus the pregnancy rate in which a fetal heart beat was present was 23.3% (95% CI=7.3 to 39.4%). OHSS was observed in three (10.0%, 95% CI=0 to 21.4%) cycles and in all cases was grade I (mild: ascites with bilateral ovarian size less than 8 cm). In all patients, OHSS symptoms resolved within 1 week with conservative management. 

There were two subjects with eight follicles ≥16 mm, and they had high estradiol levels, 3205 pg/ ml and 3493 pg/ml. These two subjects had a hyper-response, but did not get pregnant. 

**Table 1:** Baseline patient data (n=29)


Demographic	Values

Age (Y)^a^	31.0 ± 2.0
BMI (kg/m^2^)^a^	21.0 ± 2.0
Infertility^b^	
Primary	19 (65.5)
Secondary	10 (34.5)
Duration of infertility (Y)^c^	3 (1, 9)
Type of infertility^b^	
Female and male infertility*	2 (6.9)
Female infertility only	19 (65.5)
Male infertility only*	2 (6.9)
Unexplained	6 (20.7)
Causes of female infertility^b^	
Tubal factor§	5 (17.2)
Endometriosis	4 (13.8)
Ovulatory dysfunction	6 (20.7)
Others	17 (58.6)
Previous fertility treatment^b^	
None	1 (3.4)
Medication	28 (96.6)
IUI	14 (48.3)
Baseline FSH level (mIU/mL)^a^	6.1 ± 1.9
Baseline number of antral folliclesc	7 (2, 31)


*;There were four cases of male factor infertility. In these cases, the sperm concentration after washing was >1×107/ml, which was considered adequate for IUI. ^a^; Mean ± standard deviation, ^b^; Number (percentage), ^c^; Median (range), BMI; Body mass index, IUI; Intrauterine insemination, FSH; Follicle stimulating hormone, r-FSH; Recombinant FSH and §;The 5 patients with tubal factor infertility had tubal obstruction and/or adhesions which were treated prior to participation in the study.

**Table 2:** Clinical variables and outcomes of 30 cycles of ovulation induction and intrauterine insemination


Variables	Values

On the day of r-hCG administration	
Number of follicles 11-15 mm in diameter^a^	1(0,7)
Number of follicles ≥16 mm in diameter^a^	2(1,8)
E_2_level(pg/mL)^a^	898.0(60.1,3493.0)
Endometrial thickness(mm)^b^	12.0±2.4
Monofollicular development^c^	12(41.4)
Ovarian stimulation	
Total r-FSH dose(IU)^b^	1030.0±383.2
Average dailyr-FSHdose(IU)	122.5±12.6
Threshold of r-FSH(IU)^b^	131.3±23.6
Duration of r-FSH treatment(days)^b^	8.0±2.5
Outcomes	
Clinical pregnancy^c^	9(30.0%;12.6-47.4%)
Multiple pregnancy^c^	0(0%)
Number of gestational sacs with fetal heart activity^c^	7(23.3%;7.3-39.4%)
Number of gestational sacs without fetal heart activity^c^	2(6.7%;0-16.1%)
OHSS^c^	3(10.0%;0-21.4%)


Monifollicular development was defined as only one follicle with a diameter ≥16 mm. ^a^; Median (range), ^b^; Mean ± standard deviation, c; Number (percentage; 95% confidence interval), E_2_; Estradiol, OHSS; Ovarian hyperstimulation syndrome, hCG; Human chorionic gonadotropin and r-FSH; Recombinant follicle stimulation hormone.

## Discussion

The r-FSH low-dose step-up regimen described in this study was shown to be associated with a good clinical pregnancy rate (30.0%), and no multiple pregnancies were observed. In addition, though OHSS occurred in 10% of the cycles, all cases were mild and resolved with conservative management.

r-FSH, which completely lacks LH activity and extraneous human protein, has numerous advantages over prior medications (8, 10, 11). The results from a recent randomized study suggests that the use of r-FSH, as compared to urinary formulations, results in an increased clinical pregnancy rate [25.9% with follitropin alpha, 13.8% with urinary FSH and 12.5% with hepatic 3-hydroxy-3-methylglutaryl (hMG)] in IUI cycles for unexplained infertility (2). Another study, however, found that highly purified urinary FSH is as efficacious as r- FSH for ovulation induction in women with World Health Organization (WHO) group II anovulatory infertility (12) and provides a similar singleton live birth rate (15.1% with urinary FSH group vs. 15.4% with r-FSH), despite a difference in clinical pregnancy rate (17.8% with urinary FSH group vs. 21.8% with r-FSH group). Based on the clinical pregnancy rate data of the two aforementioned studies, it seems that the use of r-FSH in our study is a reasonable choice for OI/IUI.

As with prior FSH formulations, an ideal protocol of r-FSH has yet to be determined. Our protocol aimed for a monofollicular cycle and this occurred in 41.4% of the cases. It is always a challenge to determine the FSH threshold to achieve a monofollicular cycle. The lowest dose to develop a follicle has to be determined and then the optimal dose for a monofollicular cycle is determined. In this study, we set the endpoints as clinical pregnancy rate, multiple pregnancy rate, and OHSS rate rather using an endpoint of monofollicular cycles. With a starting dose of r-FSH of 112.5 IU, a few women will be hyper-responsive. These patients may do better with a lower dose (i.e. 75 IU of r-FSH), but a dose of 75 IU r-FSH might not reach the FSH threshold for most of the patients. The two subjects with eight follicles ≥16 mm exhibited a hyper-response, but did not get pregnant. They may have other infertility problems which may be addressed by *in vitro* fertilization (IVF).

Despite the fact that no multiple pregnancies in our study, there were two subjects with a hyperresponse. Thus, we cannot neglect the possibility of multiple pregnancies while utilizing this regimen into clinical practice. 

Demirol and Gurgan (2) reported an OI protocol with a daily dose of 75 IU r-FSH if the patient’s body mass index (BMI) was <25 kg/m^2^, and 150 IU if the patient’s BMI was ≥25 kg/m^2^. Balen et al. (12) treated patients with the starting dose of 75 IU r-FSH daily for 7 days, and then an increase of 37.5 IU increments according to the individual response. Chung et al. (15) compared two different r-FSH doses (150 IU vs. 100 IU every other day) with 5 days of concomitant clomiphene citrate (100 mg/day) for OI/IUI, and found that the low r-FSH dose (100 IU) resulted in a lower multiple pregnancy rate (12.5%). However, the clinical pregnancy rates reported by the authors (14.5% in the 150 IU group and 20.4% in 100 IU group) were lower than the 30% found in our study. Though the starting r-FSH dose (112.5 IU) in our study was higher than that of Balen et al. (12) and equal to the average of Demirol and Gurgan (2), the non-inferior clinical pregnancy rate in our study (30.0%) as compared to those (25.9%) and Balen et al. (21.8%), lack of multiple pregnancies and low OHSS rate suggests our protocol a viable choice for OI/IUI (2, 12).

The primary limitations of this study are the small sample size and lack of control group. A randomized controlled study comparing the rFSH low-dose step-up regimen with spontaneous/ natural cycles would be beneficial. The low-dose regimen described may decrease the overall cost; however, a cost analysis was not part of the study design. 

## Conclusion

The r-FSH low-dose step-up regimen for OI/IUI is a practical method with a low rate of complications and low risk of multiple pregnancies for younger infertile women with good pre-treatment characteristics. Further clinical studies are required to define the optimal dose of r-FSH, and whether the same regimen can be applied in aged patients with a similar outcome. 
